# Editorial: Immune correlates of protection: Insight into microbial co-infection with special emphasis on influenza-*Streptococcus pneumoniae* superinfection

**DOI:** 10.3389/fimmu.2025.1654821

**Published:** 2025-07-10

**Authors:** Tarani Kanta Barman, Avijit Dutta, Heather W. Stout-Delgado

**Affiliations:** ^1^ Department of Pathology, Galveston National Laboratory, University of Texas Medical Branch at Galveston, Galveston, TX, United States; ^2^ Amity Institute of Virology & Immunology, Amity University Uttar Pradesh, Noida, India; ^3^ Division of Pulmonary and Critical Care Medicine, Department of Medicine, Weill Cornell Medicine, Cornell University, New York, NY, United States

**Keywords:** co-infection, superinfection, immune cells, cytokines and chemokines, neutralizing antibodies, CD4 +/CD8 + T cells

Influenza virus infections are the leading cause of respiratory infections worldwide. It affects around one billion people, including 3–5 million severe cases, with 290,000 to 650,000 deaths annually (1). The major complications of influenza virus infections are secondary bacterial infections, such as *Streptococcus pneumoniae*, *Staphylococcus aureus*, group B Streptococcus, and *Hemophilus influenzae*, or fungal infections, such as Aspergillus spores, leading to exacerbation of lung pathology resulting in a considerable number of hospitalizations and deaths each year (2–5). Among these infections, a unique synergy between influenza and *Streptococcus pneumoniae* co-infection results in hyperinflammation, leading to superinfection and death. It was reported that 95% of all deaths during the 1918 pandemic were due to influenza-*Streptococcus pneumoniae* superinfection (6, 7). Experimental evidence shows that influenza infection synergistically stimulates bacterial pneumonia through dysregulated immune responses (8). Many immune cells and chemical mediators are involved in the immune response, leading to detrimental inflammation and tissue damage (9).

This Research Topic in Frontiers in Immunology under Viral Immunology, “Immune correlates of protection: Insight into microbial co-infection with special emphasis on influenza-*Streptococcus pneumoniae* superinfection,” aims to promote recent development and understanding of the cell populations and cytokines that correlate with detrimental pathology or beneficial tissue healing processes leading to disease or health. Under this topic, we collected five papers, including three original research papers and two clinical case studies. Lassnig et al., in their original study, revealed that IAV-positive pigs release vesicular neutrophil extracellular traps (NETs), which are increased in their BALF compared to IAV-negative pigs. These NET markers correlate with IAV viral load. IAV-positive BALF also enhances the growth of potential coinfecting bacteria, decreases reactive oxygen species (ROS) intensity, and enhances Actinobacillus pleuropneumoniae growth. Palani et al. investigated the genetic predisposition to IAV and bacterial coinfection in BALB/c and C57BL/6 mice. They demonstrated that genetic susceptibility to IAV/SPn coinfection was primarily attributable to the Th1/IFN-γ predisposed immune response. These findings provided a novel understanding of genetic risks and clinical preconditions for lethal post-influenza bacterial coinfection. Gou et al. found that IL-6 from the lungs prevents secondary pneumococcal infections post-influenza, offering a unique direction for research in combating complicated influenza pneumonia and secondary bacterial infections, potentially providing therapeutic avenues for influenza-*Streptococcus pneumoniae* co-infected pneumonia.

In a rare case study, Yang et al. reported *Streptococcus constellatus* coinfection that manifested as gelatinous pleural effusion in an HIV patient. They described the effusion as gelatinous and partially encapsulated; it was fully resolved with antibiotics, eliminating the need for thoracic drainage, urokinase administration, or surgery. In another case study, Feng et al. described a 72-year-old woman co-infected with four pathogens during the influenza season. They reported that early detection and timely treatment can reduce hospitalization, complication rates, and mortality. Further studies are needed to understand clinical characteristics and outcomes of co-infection, especially during the influenza season. Furthermore, additional research is needed to investigate the potential impact of SARS-CoV-2 infection on the co-occurrence of multiple respiratory pathogens.

In conclusion, the five papers on our Research Topic provide fresh scientific findings and valuable insights into bacterial-viral coinfection. The immunological correlates of protection during superinfection, the interaction of immune signaling molecules, the microbiome/colonization, and virome impacting coinfection susceptibility would remain a future area of research in bacterial-viral coinfection.
